# Comparison of Psychological Stress Levels and Associated Factors Among Healthcare Workers, Frontline Workers, and the General Public During the Novel Coronavirus Pandemic

**DOI:** 10.3389/fpsyt.2020.583971

**Published:** 2020-12-01

**Authors:** Rongrong Luan, Weidan Pu, Lilei Dai, Rui Yang, Peng Wang

**Affiliations:** ^1^Department of Psychiatry, The Affiliated Xi'an Central Hospital of Xi'an Jiaotong University, Xi'an, China; ^2^China National Clinical Research Center for Mental Health Disorders, Changsha, China; ^3^Department of Clinical Psychology, Jingmen NO.2 People's Hospital, Jingmen, China

**Keywords:** psychological stress, COVID-19, China, frontline, healthcare, association

## Abstract

**Objective:** We aimed to conduct a comparative analysis of the psychological stress experienced by healthcare workers, frontline workers, and the general public and to assess the factors associated with psychological stress in each of these groups.

**Methods:** We conducted an online survey targeting healthcare workers, frontline workers, and the general public. Psychological stress was assessed with the revised impact of event scale (IES-R). Univariable and multivariable logistic regression analyses were conducted.

**Results:** We surveyed 1,336 participants (64.6% female; mean age, 36.6). The occupation group distribution of respondents was 50.7% healthcare workers, 27.2% frontline workers, and 22.1% general public. The healthcare (23.6 ± 15.8) and frontline (23.6 ± 17.8) workers had higher IES-R scores than the general public (15.3 ± 10.6; *p* < 0.01). Poor health perception and perception of infection avoidance were associated with psychological stress in the healthcare and frontline workers, but not in the general public.

**Conclusion:** Both healthcare and frontline workers are suffering elevated psychological stress, compared to the general public, and this elevated stress may be related especially to their perceptions of their own health and infection risk. Interventions addressing these factors should be developed to alleviate psychological stress in these populations, and thus reduce their risk of mental illness pathogenesis.

## Introduction

The global health threat produced by the ongoing coronavirus disease 2019 (COVID-19) pandemic, an extraordinarily negative event, has resulted in substantial psychological stress for healthcare workers and frontline essential workers, as well as for the general public (1). Compared to severe acute respiratory syndrome, COVID-19 has had a lower mortality rate but has proven to be much more infectious (2). The difficult to control transmission of COVID-19 is having severe economic impacts (3).

The psychological stress experienced by healthcare workers, frontline essential workers, and individuals in the general public varies depending on each individual's particular situation, experiences, and the stresses that they face (4). Healthcare and frontline workers may experience heightened anxiety and depression symptoms, and thus heightened psychological stress, consequent to a constant potential exposure to COVID-19. Meanwhile, people in the general public may experience psychological stress from their inability to socialize with relatives and friends. Elucidation of the factors that are associated with psychological stress will allow governments and administrators to develop suitable interventions to manage psychological stress during this pandemic.

Although there has been some research exploring people's psychological responses to COVID-19, examinations of differences in psychological stresses between healthcare workers, frontline workers, and people in the general public have been lacking thus far. Therefore, the aims of this study were, firstly, to evaluate and compare the psychological stress experienced by healthcare workers, frontline workers and the general public, and secondly, to analyze the factors associated with psychological stress in each of the group. In doing so, we hope to be able to understand each group better and develop more targeted interventions to alleviate their psychological stress symptoms in this time of crisis.

## Methods

### Study Design

An online anonymized cross-sectional study based on the guidelines for Checklist for Reporting Results of Internet E-Surveys (CHERRIES, Table 1) (5) was conducted February 13–18, 2020 in Shaanxi province, China. The participants were recruited through a quick response code printed on posters in healthcare facilities, public venues, and community posts. We also used the WeChat platform to extend the reach of our survey. We controlled user attempts through internet protocol address verification. The ethical committee of Xi'an Central hospital approved this study and granted a waiver of informed consent before commencement of the study.

**Table 1:** Adherence to CHERRIES guidelines in the current study.

**Category**	**Checklist item**	**Described in manuscript**	**Notes**
Design	Describe survey design	Yes	
IRB (Institutional Review Board) approval and informed consent process	IRB approval	Yes	
	Informed consent	Yes	
	Data protection	No	No data protection
Development and pre-testing	Development and testing	No	Tested and adjusted
Recruitment process and description of the sample having access to the questionnaire	Open survey vs. closed survey	No	Open survey
	Contact mode	Yes	
	Advertising the survey	Yes	
Survey administration	Web/E-mail	Yes	
	Context	Yes	
	Mandatory/voluntary	No	Voluntary
	Incentives	No	No incentives
	Time/date	Yes	
	Randomization of items or questionnaires	No	Not randomized
	Adaptive questioning	No	No adaptive questioning
	Number of items	No	61 items
	Number of screens (pages)	No	2 pages
	Completeness check	No	Yes
	Review step	No	Yes
Response rates	Unique site visitor	No	3,778
	View rate (ratio of unique survey visitors/unique site visitors)	No	62.6% (2,365/3,778)
	Participation rate (ratio of unique visitors who agreed to participate/unique first survey page visitors)	No	100%
	Completion rate (ratio of users who finished the survey/users who agreed to participate)	No	56.5% (1,336/2,365)
Preventing multiple entries from the same individual	Cookies used	No	No
	IP check	No	Yes
	Log file analysis	No	No
	Registration	No	No need
Analysis	Handling of incomplete questionnaires	No	Analyzed only completed surveys
	Questionnaires submitted with an atypical timestamp	No	No timestamp
	Statistical correction	No	No correction

### Subjects

We included participants who were at least 18 years old; juveniles were excluded because a self-report survey was used. Participant groups included healthcare workers, front line workers (cleaners, police, delivery personnel, administrators whose work is directly involved with the pandemic, security personnel, mortuary workers, etc.), and the general public.

### Data Collection

We collected sociodemographic, clinical, and patient-reported outcome measure (PROM) data. Sociodemographic data included gender, age, monthly income, and perceived personal risk. Clinical data included the presence of mental illnesses and chronic diseases. Five target questions were developed based on former studies of pandemics, such as SARS [e.g., (6, 7)], advice from psychiatry and infectious diseases experts, and our own clinical observations. The target questions are related principally to increased workload, reduced sleep quality, personal health perception, infection avoidance perception, and confidence. These five questions were answered on a 5-point Likert scale (no change, slight changes, moderate changes, severe changes, and extremely severe changes). We administered the following PROM instruments: Impact of Event Scale-Revised (IES-R), Patient Health Questionnaire-9 (PHQ-9), and General Anxiety Disorder-7 (GAD-7).

#### IES-R

The IES-R was designed to measure the impact of events on one's psychological stress level (8). It has three subscales: intrusion, avoidance and hyperarousal. IES-R scores range from 0 to 88, with higher scores signifying a greater stress impact. The scale has been shown to be valid and reliable in the Chinese population (8).

#### PHQ-9

The PHQ-9 is a scale used to detect the presence and severity of depression symptoms (9). PHQ-9 scores range from 0 to 27, with higher scores indicating more severe depression symptoms. The PHQ-9 has been found to be valid and reliable for Chinese respondents (9).

#### GAD-7

The GAD-7 is a scale that assesses the presentation and severity of anxiety symptoms (10). GAD-7 scores range from 0 to 21, wherein higher scores indicate greater anxiety. The scale has been found to be valid and reliable in the Chinese population (10).

### Statistical Analysis

Descriptive statistics are reported as frequency (percentage) or mean ± standard deviation as appropriate. Shapiro-Wilk test was used to assess dataset normality. Chi square or Fisher's exact tests were used to analyze categorical data as appropriate. Analyses of variance or Kruskal Wallis tests were used to analyze continuous data as appropriate. To assess factors associated with psychological stress, we dichotomized IES-R scores into >35 and ≤35 was conducted. Variables with *p* < 0.1 in univariable logistic regression were included in subsequent multivariable logistic regression. *P* < 0.05 were considered statistically significant. The data were analyzed in SPSS 22.0 (IBM, Armonk, NY).

## Results

Survey data collected from 1,336 participants were analyzed. The age and gender distributions of the study sample are reported for the whole cohort and compared across study groups (i.e., healthcare worker, frontline worker, or general public respondent) in Table 2. Overall, the population sample consisted of 678 (50.7%) healthcare workers, 363 (27.2%) frontline workers, and 295 (22.1%) general public respondents. The distributions of these respondents with respect to job categories within each study group and PROM scores by study group are also reported and compared in Table 1. Notably, we observed significantly higher IES-R scores for the healthcare and frontline worker groups than for the general public.

**Table 2:** Characteristics of the participants.

**Characteristic**	**Total *N* = 1,336**	**Healthcare workers *N* = 678**	**Frontline workers *N* = 363**	**General public *N* = 295**	***P***
Age	36.7 ± 9.1	35.6 ± 7.8	37.1 ± 9.0	38.5 ± 11.3	<0.01
Female gender	863 (64.6%)	554 (81.7%)	142 (39.1%)	167 (56.6%)	<0.01
Occupation					
**Healthcare workers**					
Doctors	242 (18.1)	242 (35.6)	–	–	
Nurses	188 (14.1)	188 (27.7)	–	–	
Allied health	166 (12.4)	166 (24.4)	–	–	
Administrators and operation workers	82 (6.1)	82 (12.0)	–	–	
**Frontline workers**					
Police	115 (8.6)	–	115 (31.6)	–	
Civil Service	146 (10.9)	–	146 (40.2)	–	
Delivery personnel	55 (4.1)	–	55 (15.1)	–	
Cleaners	41 (3.1)	–	41 (11.2)	–	
Other	6 (0.4)	–	6 (1.6)	–	
**General public**					
Teachers	87 (29.5)	–	–	87 (29.5)	
Employees	65 (22.0)	–	–	65 (22.0)	
Self-employed	49 (16.6)	–	–	49 (16.6)	
Students	36 (12.2)	–	–	36 (12.2)	
Other	58 (19.7)	–	–	58 (19.7)	
**Target question**					
Exposure risk	2.84 ± 1.04	3.39 ± 0.60	3.13 ± 0.38	1.21 ± 0.66	<0.01
Workload	2.18 ± 1.24	2.30 ± 1.18	2.42 ± 1.39	1.62 ± 0.97	<0.01
Sleep quality	1.91 ± 1.06	1.98 ± 1.03	2.04 ± 1.16	1.58 ± 0.92	<0.01
Health perception	3.58 ± 0.96	3.53 ± 0.93	3.49 ± 1.04	3.82 ± 0.89	<0.01
Infection avoidance perception	3.53 ± 1.13	3.33 ± 1.07	3.62 ± 1.18	3.86 ± 1.11	<0.01
Confidence	4.46 ± 0.82	4.36 ± 0.87	4.51 ± 0.79	4.64 ± 0.67	<0.01
**IES-R**					
Full scale	21.8 ± 15.8	23.6 ± 15.8	23.6 ± 17.8	15.2 ± 10.6	<0.01
Intrusion subscale	8.1 ± 6.2	8.9 ± 6.2	8.6 ± 7.0	5.6 ± 4.2	<0.01
Avoidance subscale	7.3 ± 6.0	7.7 ± 5.9	7.8 ± 6.6	5.5 ± 4.6	<0.01
Hyperarousal subscale	6.4 ± 5.2	7.0 ± 5.2	7.2 ± 5.7	4.2 ± 3.4	<0.01
PHQ-9	4.9 ± 5.5	5.6 ± 5.6	5.9 ± 6.2	2.2 ± 2.3	<0.01
GAD-7	3.9 ± 4.8	4.5 ± 4.8	4.9 ± 5.5	1.3 ± 1.7	<0.01

*Target question response ranges were 1–5. IES-R (full scale), PHQ-9, and GAD-7 scores ranges were 0–88, 0–27, and 0–21, respectively*.

As reported in detail in Table 3, our univariable logistic regression analyses of factors that may be associated with psychological stress indicated that female gender, high workload, poor sleep quality, poor health perception, low perception of infection avoidance, low confidence, high PHQ-9 score, and high GAD-7 score were associated with greater psychological stress in our survey participants. Comparing between the three study groups, high risk exposure was associated with elevated psychological stress specifically among frontline workers. Meanwhile, poor health perception, low perception of infection avoidance, and low confidence were associated with elevated psychological stress among healthcare and frontline workers, but not among general public respondents.

**Table 3:** Univariable analysis of factors associated with IES-R scores.

**Characteristic**	**Total**	**Healthcare workers**	**Firstline workers**	**General public**
Age	1.00 (0.98, 1.01)	1.00 (0.97,1.01)	1.01 (0.98, 1.04)	1.01 (0.95, 1.07)
Female gender	1.73 (1.27, 2.36)^**^	1.64 (0.98, 2.75)^*^	1.91 (1.17, 3.12)^**^	2.76 (0.56, 13.50)
**Target question**				
Exposure risk	1.84 (1.57, 2.17)^**^	1.12 (0.84, 1.50)	2.91 (1.63, 5.17)^**^	1.453 (0.71, 2.96)
Workload	1.89 (1.68, 2.11)^**^	1.93 (1.64, 2.26)^**^	1.53 (1.28, 1.82)^**^	2.22 (1.35, 3.63)^**^
Sleep quality	2.57 (2.24, 2.94)^**^	2.71 (2.23, 3.29)^**^	2.31 (1.85, 2.90)^**^	2.31 (1.43, 3.75)^**^
Health perception	0.55 (0.48. 0.64)^**^	0.61 (0.50, 0.74)^**^	0.54 (0.42, 0.69)^**^	0.63 (0.31, 1.27)
Perception of infection avoidance	0.53 (0.47, 0.61)^**^	0.53 (0.44, 0.63)^**^	0.54 (0.42, 0.69)^**^	0.68 (0.40, 1.15)
Confidence	0.51 (0.44, 0.60)^**^	0.53 (0.44, 0.65)^**^	0.54 (0.43, 0.67)^**^	1.05 (0.38, 2.89)
PHQ-9	1.27 (1.23,1.31)^**^	1.23 (1.18. 1.28)^**^	1.26 (1.19, 1.33)^**^	1.45 (0.71, 2.96)^**^
GAD-7	1.40 (1.34, 1.45)^**^	1.35 (1.28, 1.42)^**^	1.39 (1.30, 1.50)^**^	1.88 (1.33, 2.67)^**^

*Data are presented as ORs (95% CIs)*.

* *p < 0.1*,

** *p < 0.05, using univariate logistics regression*.

*Target question response ranges were 1–5. PHQ-9 and GAD-7 scores ranges were 0–27 and 0–21, respectively*.

As reported in Table 4, the following factors were found to be associated with psychological stress in our multivariable logistic regression: high workload, poor sleep quality, poor health perception, low perception of infection avoidance, high PHQ-9 score, and high GAD-7 score were associated with higher psychological stress levels in our survey participants. High workloads and high PHQ-9 scores were associated with higher psychological stress in healthcare workers, but not in first line workers or in the general public. Poor health perception and a low perception of infection avoidance were associated with higher psychological stress in healthcare and frontline workers, but not in the general public.

**Table 4:** Results of multivariable analysis of factors associated with IES-R scores.

**Characteristic**	**Total**	**Healthcare workers**	**Firstline workers**	**General public**
Female gender	1.50 (0.98, 2.30)	1.23 (0.63, 2.39)	1.87 (9.14, 3.83)	NIL
**Target question**				
Exposure risk	1.11 (0.89, 1.39)	NIL	1.56 (0.64, 3.75)	NIL
Workload	1.33 (1.13, 1.56)^**^	1.35 (1.10, 1.67)^**^	1.20 (0.91, 1.59)	1.19 (0.93, 1.53)
Sleep quality	1.50 (1.24, 1.82)^**^	1.56 (1.21, 2.03)^**^	1.47 (1.04, 2.07)^*^	1.39 (1.01, 1.92)^*^
Health perception	1.51 (1.19, 1.90)^**^	1.51 (1.12, 2.03)^**^	1.52 (1.00, 2.29)^*^	NIL
Perception of infection avoidance	0.67 (0.56, 0.81)^**^	0.63 (0.50, 0.80)^**^	0.71 (0.51, 0.98)^*^	NIL
Confidence	0.88 (0.71, 1.10)	0.82 (0.63, 1.07)	0.95 (0.61, 1.46)	NIL
PHQ-9	1.09 (1.03, 1.15)^**^	1.09 (1.02, 1.17)^**^	1.08 (0.99, 1.18)	1.05 (0.97, 1.13)
GAD-7	1.23 (1.16, 1.31)^**^	1.17 (1.08, 1.27)^**^	1.27 (1.15, 1.40)^**^	1.30 (1.18, 1.43)^**^

*Data are presented as ORs (95% CIs)*.

* *p < 0.05*,

** *p < 0.01, multivariate logistics regression*.

*Target question response ranges were 1–5. PHQ-9, and GAD-7 scores ranges were 0–27 and 0–21, respectively*.

## Discussion

Our study revealed that healthcare and frontline workers experienced greater psychological stress than individuals in the general public. Overall, we found that higher psychological stress was associated with the following six factors: a high workload; poor sleep quality; poor health perception; low perception of infection avoidance; high PHQ-9 score; and high GAD-7 score. We observed dissociations among our occupation study groups, with poor health perception and a low perception of infection avoidance being associated with higher psychological stress in healthcare and frontline workers, but not in the general public. This study was the first to examine psychological stress and its associated factors in three different groups of participants with a large sample size in a country undergoing a serious COVID-19 epidemic.

Greater psychological stress among healthcare and frontline workers than in the general public is likely due to their direct contact with patients and colleagues that have become ill with COVID-19 (4). Healthcare and frontline workers may also experience elevated anxiety in relation to a constant pressure to perform their duties in the face of adversity (4). These findings are consistent with results obtained during other epidemics (11). Potential interventions can be developed to alleviate these psychological stresses in healthcare and frontline workers, and thus help to reduce the risk of mental illness pathogenesis in these populations.

Although our findings indicating that healthcare and frontline workers who showed higher anxiety and depression symptom levels were also suffering a greater psychological stress load are consistent with prior findings, the present results were the first to link psychological stress to individuals' perceptions of infection avoidance and personal health (12). These findings underscore the importance firstly of providing adequate personal protective equipment together with appropriate training on their use and secondly of ensuring that only healthy staff are deployed are critically important measures for supporting the psychological status of our healthcare workers (13).

Furthermore, this study is the first to reveal the impact of COVID-19 on the psychological stress of frontline workers. Although delivery personnel and cleaning staff are not healthcare personnel, they play a very important role in supporting the ongoing and optimal functioning of healthcare workers and of the general public. Because of the nature of their work, they are also exposed to COVID-19 frequently and often lack appropriate personal protective equipment, which is more available to healthcare workers. Therefore, frontline workers are in need of greater protective measures to support them psychologically so that they can remain in their jobs helping their fellow citizens during the ongoing COVID-19 pandemic (14, 15).

Our findings showing that anxiety and depression symptoms, as measured with the GAD-7 and PHQ-9, respectively, were associated with psychological stress across all three groups of participants are consistent with Moyer et al.'s previous research linking GAD-7 and PHQ-9 scores with acute stress (16). Thus, these PROM instruments may be useful for assessing the effects of interventions developed to alleviate psychological stress in times of crisis (16).

There are some notable limitations of our study. First, because we did not ensure a randomized sample, there may be some bias associated with convenience sampling. However, given that we are in the midst of an ongoing pandemic, a randomized sample was not feasible. Second, the use of an online platform, namely WeChat, was likely to have had a selective sampling influence. In this regard, it should be note that WeChat is one of the most popular online applications currently in use within China. Indeed, virtually all retailers use WeChat for financial transactions in support of cashless commerce. Hence, it is an online application that virtually all Chinese people should be familiar with. Last, because we employed a cross-sectional study design, we cannot make conclusions about causality in the detected associations.

In conclusion, we found that healthcare and frontline workers experienced greater psychological stress than people in the general public. Psychological stress was found to be related to respondents' perception of their own health and risk of being infected among both healthcare and frontline workers, but not among participants in the general public. This study provides useful information for policy decision makers with respect to the provision of supports to healthcare and frontline workers in countries experiencing the challenges of a pandemic.

## Data Availability Statement

The raw data supporting the conclusions of this article will be made available by the authors, without undue reservation.

## Ethics Statement

The studies involving human participants were reviewed and approved by The ethical committee of Xi'an Central hospital approved this study. The ethics committee waived the requirement of written informed consent for participation.

## Author Contributions

RL and PW designed the study, wrote the protocol, and collected the data. RL and LD analyzed the data. RL and WP drafted the manuscript. RY and PW revised the manuscript. All the authors commented on the manuscript and have approved the final manuscript.

## Conflict of Interest

The authors declare that the research was conducted in the absence of any commercial or financial relationships that could be construed as a potential conflict of interest.
